# The Application of a Physiologically Based Toxicokinetic Model in Health Risk Assessment

**DOI:** 10.3390/toxics11100874

**Published:** 2023-10-21

**Authors:** Mengting Chen, Ruihu Du, Tao Zhang, Chutao Li, Wenqiang Bao, Fan Xin, Shaozhang Hou, Qiaomei Yang, Li Chen, Qi Wang, An Zhu

**Affiliations:** 1 Key Laboratory of Ministry of Education for Gastrointestinal Cancer, School of Basic Medical Sciences, Fujian Medical University, Fuzhou 350108, China; 2 Department of Toxicology, School of Public Health, Peking University, Beijing 100191, China; 3 Department of Pathology, School of Basic Medical Sciences, Ningxia Medical University, Yinchuan 750004, China; 4 Department of Gynecology, Fujian Maternity and Child Health Hospital (Fujian Obstetrics and Gynecology Hospital), Fuzhou 350001, China; 5 Key Laboratory of State Administration of Traditional Chinese Medicine for Compatibility Toxicology, Beijing 100191, China; 6 Beijing Key Laboratory of Toxicological Research and Risk Assessment for Food Safety, Beijing 100191, China

**Keywords:** compartmental model, exposure routes, PBTK model, health risk assessment

## Abstract

Toxicokinetics plays a crucial role in the health risk assessments of xenobiotics. Classical compartmental models are limited in their ability to determine chemical concentrations in specific organs or tissues, particularly target organs or tissues, and their limited interspecific and exposure route extrapolation hinders satisfactory health risk assessment. In contrast, physiologically based toxicokinetic (PBTK) models quantitatively describe the absorption, distribution, metabolism, and excretion of chemicals across various exposure routes and doses in organisms, establishing correlations with toxic effects. Consequently, PBTK models serve as potent tools for extrapolation and provide a theoretical foundation for health risk assessment and management. This review outlines the construction and application of PBTK models in health risk assessment while analyzing their limitations and future perspectives.

## 1. Introduction

Toxicokinetics (TK) is an interdisciplinary field that integrates the principles and methodologies of pharmacokinetics and toxicology. It plays a crucial role in understanding the absorption, distribution, metabolism, excretion, and toxicity (ADMET) characteristics of xenobiotics in both human and animal models, considering various exposure routes, doses, and frequencies. TK data enables the calculation of kinetic parameters and facilitates adjustments to experimental designs. By establishing the relationship between dose-exposure and exposure-toxicity, TK allows for the assessment of toxicity effects, mechanisms in target organs and tissues, and the associated risks to human health.

Traditional TK studies often involve doses higher than those used in clinical pharmacokinetic research, resulting in saturation of dissolution, absorption, and metabolism processes and leading to nonlinear kinetics models [1,2]. Therefore, the bioavailability, half-life, apparent volume of distribution, clearance, and other parameters calculated from low-dose pharmacokinetic studies may not be applicable to high-dose toxicokinetic investigations. To enable reliable interspecific extrapolation, dose extrapolation, and in vivo-in vitro correlation, it is essential to systematically consider dose-response and dose-effect relationships in TK studies. After a satisfactory evaluation, the PBTK model is utilized to extrapolate and compute internal doses, thereby enhancing the dose-response relationship in toxicity testing and risk assessment [3]. The European Human Biomonitoring Initiative effectively employed the PBTK model to evaluate the association between chemical exposure and observed HBM data [4]. In the drug review process conducted by the United States Food and Drug Administration (FDA), submitted PBTK models from drug developers were assessed for adequacy at different stages of drug development to achieve desired objectives. Previous studies had solely focused on developing rat-based PBTK models to assess risks associated with oral exposure to diethyl phthalate (DEP), and Hu et al. [5] developed a human dermal pathway-specific model for assessing health risks when exposed to DEP, which suggested that dermal exposure to DEP posed a greater risk to human health compared with oral exposure.

In general, four conventional methods are employed for the calculation of pharmacokinetic or toxicokinetic parameters, including the compartmental model, statistical moment analysis of the noncompartmental model, population pharmacokinetics or toxicokinetic analysis, and the physiologically based pharmacokinetics or toxicokinetic (PBPK/PBTK) model. However, the first three methods do not consider physiological characteristics and, thus, can only reflect concentration changes over time in specific tissues or organs, lacking the ability to analyze the distribution and metabolism of xenobiotics simultaneously. As a result, these methods fail to extrapolate prediction results across different species, exposure routes, and doses [1,6,7]. In contrast, PBTK models represent a more complex form of compartmental models, where tissue and organ entities act as interconnected compartments through blood circulation. The parameters in PBTK models possess physiological significance, such as blood flow volume and tissue volume, enabling the simulation of xenobiotic absorption, distribution, metabolism, and excretion in various tissues or organs, thereby facilitating extrapolation. This review primarily focuses on constructing and applying the PBTK model in health risk assessments while analyzing its limitations and future perspectives.

## 2. The Construction of the PBTK Model

**Figure 1:**
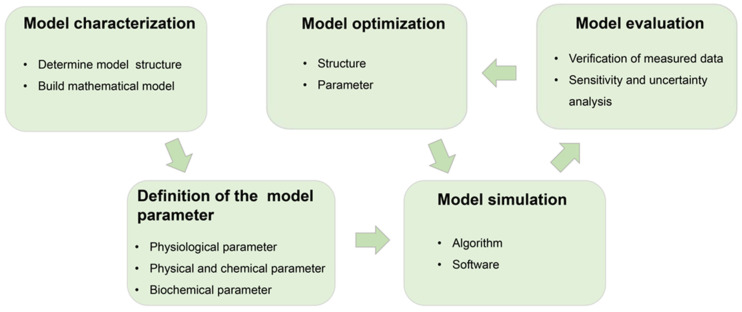
The diagram for the construction of the PBTK model.

The construction of a PBTK model typically involves five steps, as illustrated in Figure 1: (1) model characterization, (2) definition of the model parameters, (3) model simulation, (4) model evaluation, and (5) model optimization [1,7,8,9].

### 2.1. Model Characterization

#### 2.1.1. Determination of the Model Structure

**Figure 2:**
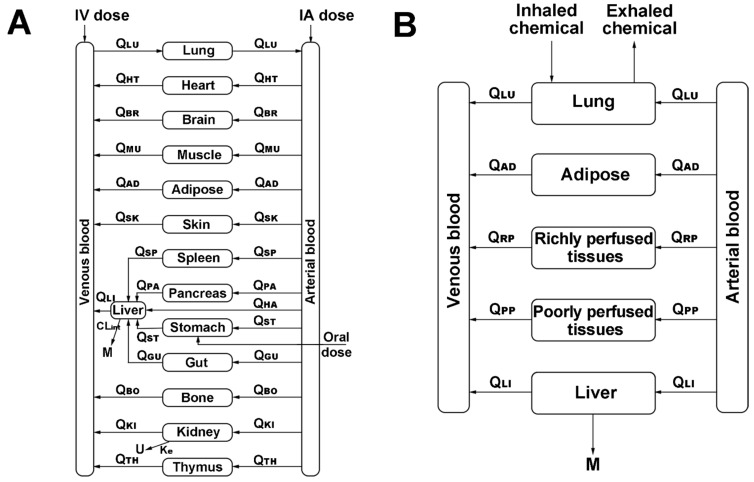
The whole PBTK model (**A**) and semi-PBTK model (**B**) of humans. IV, intravenous injection; IA, arterial injection; Q, blood flow; M, metabolites; U, urine; CLint, intrinsic clearance; Ke, excretion rate. The subscripts LU, HT, BR, MU, AD, SK, SP, PA, HA, ST, GU, BO, KI, TH, RP, PP, and LI refer to lung, heart, brain, muscle, fat, skin, spleen, pancreas, hepatic artery, stomach, gut, bone, kidney, thymus, richly perfused tissue, poorly perfused tissue, hepatic vein, respectively.

The structure of the PBTK model is based on physiological and anatomical considerations; it can be either a whole-body PBTK model or a semi-PBTK model, depending on the complexity required. In the whole-body PBTK model (Figure 2A), each organ or tissue is considered as an independent compartment, connected through blood flow to others [10]. In the semi-PBTK model (Figure 2B), major organs like blood, liver (the main metabolic organ), and kidneys (the main excretory organ) are separate compartments. Other organs not relevant to the study are grouped into one compartment, simplifying the structure and reducing the number of parameters for improved parameter fitting accuracy. Figure 2B illustrates a common human semi-PBTK model with a respiratory exposure route. In this model, organs or tissues not directly relevant to the study are categorized as richly perfused tissues (e.g., brain, heart, spleen, intestines, kidneys, adrenal glands, thyroid, lungs, and bone marrow) or poorly perfused tissues (e.g., muscle and skin) [11].

During the modeling process, consideration must be given to which organs or tissues should be included. Firstly, the PBTK model includes compartments for blood, metabolic organs (such as the liver), and excretory organs (such as the kidneys), as they are closely associated with toxicokinetic studies. Secondly, it is necessary to determine whether other organs or tissues should be included in the chemical ADMET processes. For example, in the case of oral administration, compartments for the stomach and gut should be added. For lipophilic chemicals with high octanol-water partition coefficients (log Kow > 3), an independent compartment for adipose tissue should be included. Thirdly, adherence to the parsimony principle is crucial [9]. As model complexity increases, more data are required for fitting and validation purposes. In the field of chemical toxicology, greater attention has been focused on target organs or tissues such as the liver and kidney, while fewer studies have explored other organs or tissues comprehensively. Due to insufficient data availability that can support a complex PBTK model effectively, it is essential to simplify the model as much as possible in order to enhance its predictive reliability.

#### 2.1.2. Building Mathematical Equations

After determining the model structure, mathematical equations were established to describe the ADME processes of xenobiotics in each organ or tissue. The establishment of the model should follow the principle of mass equation [7,9,12]:

(1) $$\frac{{dA}_{t}}{dt}=A_{t,in}-A_{t,out}-A_{t,e}-A_{t,m}$$

where dA_t_/dt is the amount if change of xenobiotics in a compartment per unit time; A_t,in_ is the amount of entry into the compartment; A_t,out_ is the quantity leaving the compartment; A_t,e_ is the amount excreted by the compartment; and A_t,m_ is the amount of compartment metabolism.

In the PBTK model, the absorption process of xenobiotics by organs or tissues obeys the simple diffusion principle in Fick’s law [1,11]. Based on the distribution characteristics of xenobiotics in organs or tissues, PBTK models can be categorized into three types:

(1) Perfusion-limited model: in this model, xenobiotic concentrations in organs, tissues, and blood reach instant equilibrium without concentration differences. Organs, tissues, and blood are considered as a single compartment with a homogeneous distribution of xenobiotics. The only limiting factor for xenobiotic distribution is blood flow velocity [12,13]. This model is suitable for small lipid-soluble molecules that can readily cross membrane barriers and when organs or tissues are small-sized with high blood flow [7,14,15];
(2) Permeability-limited model: in this model, xenobiotics penetrate organs or tissues through membrane barriers, resulting in a concentration gradient between organs or tissues and blood. Depending on the number of membrane barriers, the permeability-limited model can have two or three sub-compartments. This model is suitable for molecules with polarity or large molecular weight [14,15];
(3) Dispersion model: in this model, xenobiotics are distributed in organs or tissues with a gradient, and the degree of dispersion is evaluated using the dispersion coefficient (D_N_). A higher D_N_ indicates a greater dispersion of xenobiotics in organs or tissues. When D_N_ approaches infinity, the dispersion model is similar to the perfusion-limited model [16,17,18,19,20,21]. This model is suitable for xenobiotics with high hepatic clearance [8].

The metabolism of xenobiotics can be described as first-order kinetic, second-order kinetic, and saturation process [11,15]. Table 1 provides commonly used mathematical equations to describe the ADME processes in PBTK models [15].

### 2.2. Definition of Model Parameters

As shown in Table 2, the PBTK model mainly contains physiological, physicochemical, and biochemical parameters. The physiological parameters include animal body weight, cardiac output, blood flow through an organ or tissue, the volume of the organ or tissue, and so on [22,23,24,25]. The physiological parameters are obtained from the literature, depending on the specific study objectives, or determined through experiments [26,27,28,29]. Physiological parameters can be influenced by factors such as age, sex, and diseases.

Physicochemical parameters refer to the blood-air distribution coefficient and tissue-blood distribution coefficient, which represent the concentration ratio of xenobiotics in different carriers at steady state and vary with the properties of the xenobiotics. For xenobiotics with insufficient documented physicochemical parameters, data can be obtained through three approaches. Firstly, in vivo experiments can provide distribution coefficients based on steady-state toxicokinetic data from repeated administration or dynamic data from single intravenous administration [11,30,31,32,33,34]. Secondly, in vitro experiments such as vial equilibration [35,36,37,38,39,40,41,42,43,44], equilibrium dialysis [45,46,47], ultrafiltration [46], and others have been reported for determining distribution coefficients. Thirdly, in silico approaches involve computation and simulation methods that determine distribution coefficients based on structural characteristics and physicochemical properties of xenobiotics [48,49,50,51,52,53,54,55,56,57,58].

Biochemical parameters include the absorption rate constant, maximum velocity, Michaelis constant, etc. These parameters can be calculated using plasma or tissue xenobiotic concentration versus time profiles and excretion data from in vivo [59] or in vitro [60,61,62,63,64,65] experiments. They can also be obtained through computation and simulation using quantitative structure–activity relationship models based on the molecular structure of xenobiotics [66,67,68,69]. It is important to note that metabolic parameters obtained from in vitro experiments cannot be directly used in an in vivo PBTK model. Appropriate data transformation should be performed, considering the metabolic differences between in vitro and in vivo studies [11,70,71]. In order to identify the most sensitive parameters within existing mammalian models, Schneckener et al. [72] extrapolated a validated rabbit PBTK model to six other mammalian species using established species-specific models. By calculating sensitivity across various mammalian species, different compounds, and diverse administration routes, only 5.4% (*n* = 33) parameters had sensitivity values over 0.4.

### 2.3. Model Simulation

PBTK model simulation involves generating predictive curves of toxicokinetics that fit with measured data by solving ordinary differential equations through mathematical calculations and software.

#### 2.3.1. Algorithm

Common algorithms used to solve ordinary differential equations are Euler, Gear, Runge-Kutta, and predictor-corrector. These algorithms follow the principle:

(14) New value=old value+slope×dt

where dt is the calculated step value, and slope is the first derivative of the equation of a curve at a given point. For a particular compartment in the PBTK model,

(15) $$A_{t,1}=A_{t,0}+({dA}_{t}/dt)\times dt$$

where A is the total amount of xenobiotics, and the subscripts t,0 and t,1 represent the start and end times of each calculation, respectively.

The Euler is the most frequently used algorithm to solve ordinary differential equations in the PBTK model [73,74]. The error generated by the Euler algorithm is proportional to the square of the step value (dt^2^), so a smaller dt^2^ is needed to reduce this error, although it requires more calculation time. In addition, the Euler algorithm is not applicable to stiff system solutions, for which the Gear algorithm is recommended due to its greater stability [11].

#### 2.3.2. Software

Existing software for PBTK modeling and analysis can be divided into two categories; the first is general mathematical and engineering modeling software, such as MATLAB, Berkeley Madonna, R, and acslX. These software packages require users to build a model framework and write corresponding codes, necessitating high modeling and programming skills. The advantage of these software packages is their high flexibility [75], allowing users to construct customized PBTK models based on their needs and perform complex analyses such as uncertainty analysis, Monte Carlo analysis, and Markov chain Monte Carlo (MCMC) analysis. The second is the professional PBTK and modeling software, such as Simcyp, PK-Sim, GastroPlus, Cloe PK, PKQuest, etc. This software is user-friendly and requires minimal modeling and programming skills. Users can either combine various components of the model or directly use built-in models provided by the software. Some software packages also integrate functions for predicting the physicochemical properties of xenobiotics. These software options are suitable for researchers who are not interested in computation and programming or lack modeling experience [7,8,9,76,77]. Mahdi et al. [78] investigated a suitable solvent for subcutaneous delivery of rifampicin through in silico predictions by GastroPlus, which reduced the cost and time required for formulation development by utilizing in vivo data to simulate in vitro findings. Hanke et al. [79] developed a rosuvastatin model using the open-source PBTK software PK-Sim, incorporating plasma, urine, and feces data of rosuvastatin, positron emission tomography measurements of tissue concentrations, and seven different rosuvastatin drug–drug interaction studies. The PK-Sim expression database provided the relative expression in different organs by analyzing drug transporters and metabolizing enzymes. However, there are certain limitations when using software to predict drug metabolism. These software have a high threshold and need users to receive sufficient professional knowledge on the data and parameters that are to be used in the implementation. The establishment of a PBTK model needs numerous drug parameters, so further validation and optimization of the constructed model are essential to improve its accuracy.

### 2.4. Model Evaluation

A constructed PBTK model should undergo evaluation under various scenarios, particularly its ability to predict experimental data not used in the modeling process. Additionally, uncertainty, variability, and sensitivity analyses are performed [7,11].

#### 2.4.1. Verification of Measured Data

**Figure 3:**
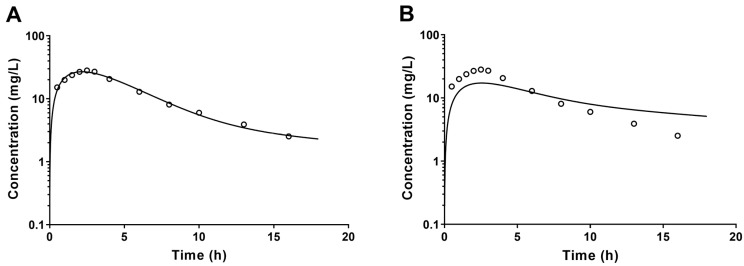
In PBTK models, the left panel (**A**) is superior to the right panel (**B**) on the consistency between the predicted value (solid line) and the measured value (hollow circle) by visual inspection.

The predictive performance of the PBTK model is evaluated through visual inspection, statistical tests, and discrepancy indices by comparing predicted values to measured values [1,11,80,81]. Based on visual inspection, the most commonly used method, Figure 3A exhibits superior predictive ability compared to Figure 3B due to the logarithmic scale typically used for the concentration versus time curve.

In the context of PBTK model validation, statistical hypothesis tests and discrepancy tests are not applicable because the null hypothesis assumes that the model is identical to the actual biological system, which is not realistic [80]. Classical statistical methods such as Mann-Whitney, two-sample χ^2^, and two-sample Kolmogorov are also not suitable for assessing consistency between predicted and measured values due to the self-correlation of concentration values at different times in pharmacokinetics [11]. To assess the predictive ability of the PBTK model, linear regression analysis can be performed to compare the predicted and measured values. A high prediction ability is indicated when the intercept of the regression equation is close to 0 and the slope and correlation coefficient are close to 1 [10,77]. Another method for assessing the predictive ability of the PBTK model is to calculate the chi-square χ^2^ as Equation (16). This approach is applicable when there are multiple measured values at the same time point. Peters used this method in an overall PBTK model to evaluate the model fitting [82]. An χ^2^ value close to 1 indicates better model fitting and prediction performance. The Akaike information criterion (AIC) and Bayesian information criterion (BIC) are commonly used for assessing predictive ability [83,84]. AIC and BIC are calculated using Equations (17) and (18), respectively. Smaller values of AIC and BIC indicate a better model fit. When there is a significant difference between the fitted model and the real model, it is primarily reflected in the likelihood function term −2In(L). When the difference in the likelihood function is not significant, the penalty term 2k, accounting for the number of model parameters, becomes influential. Models with better fit and fewer parameters are preferred as the increase in the number of parameters leads to increased 2k and AIC values. The penalty term for BIC is larger than that for AIC, taking into account the number of observations. This prevents excessive model complexity when there is a large number of observations. The last method is to calculate the fold error (FE), as Equation (19). Firstly, the noncompartmental analysis was performed to obtain the peak concentration, peak time, area under the curve, and other parameters of the observations. Then, the fitting values or predicting values of the above parameters were obtained from corresponding models. Due to the different laboratory conditions, detection methods, and other confounding factors, the measured value and the predicted value cannot be consistent completely, and the model is acceptable when the FE ≤ 2 [80].

(16) $$\begin{array}{c} \chi^{2}=\frac{1}{n}\sum_{i=1}^{n}(\frac{\Delta_{i}^{2}}{\sigma_{i}^{2}})\; \end{array}$$

In Equation (16), n is the number of observations, Δ is the residual, i.e., the difference between the measured and predicted values, and σ is the standard deviation of the measured values at the same time point.

(17) $$\begin{array}{c} \mathrm{AIC}=2k-2\mathrm{In}\left(L\right)\; \end{array}$$

(18) $$\begin{array}{c} \mathrm{BIC}=\mathrm{In}\left(n\right)\cdot k-2\mathrm{In}\left(L\right)\; \end{array}$$

In Equations (17) and (18), k is the number of model variables, L is the likelihood function, and n is the number of observations.

(19) $$\begin{array}{c} \mathrm{FE}=10^{\left|log\left(\frac{Simulated}{Observed}\right)\right|}\; \end{array}$$

#### 2.4.2. Uncertainty Analysis

The PBTK model is constructed according to the physiological and anatomical features artificially, and these parameters are obtained from in vitro and in vivo experiments or in silico predictions. An illogical model structure or inaccurate parameters will result in an inaccurate prediction [7,11]; therefore, the uncertainty analysis is performed to assess the impact of parameter errors on model prediction results. Three methods are used for uncertainty analysis; the first is the Monte Carlo simulation, which calculates an output parameter value by randomly selecting input parameter values fitting specific probability distribution. Cox [85] employed Monte Carlo uncertain analysis within a PBTK model to re-evaluate the risk assessments of benzene metabolism in animals and humans, and the probability distribution of the entire dose–response function for benzene-induced tumors in male B6C3F1 mice was quantified. The second method is probability-bound analysis; in this approach, input parameters are systematically varied from minimum to slightly above minimum, then set at the normal value, followed by slightly below the maximum, and finally at the maximum of the distribution. Concurrently, the corresponding output parameters are observed, representing extreme values of parameter variation. The third is the Fuzzy simulation, in which the parameters are fuzzed after input, and the output is obtained by fuzzy rules. If the distributions of uncertain parameters are known, the Monte Carlo simulation method can be used [86,87,88,89,90]. Otherwise, the fuzzy simulation is suggested [91,92,93]. If only the maximum and minimum values of the parameters are known, the probability-bound analysis is suggested [11,94]. Through the uncertainty analysis, the impact of input parameters on the model is elucidated and improves the prediction ability of the model.

#### 2.4.3. Variation Analysis

When constructing the PBTK model for population studies, the physiological and biochemical parameters are variable due to individual differences. The variation analysis is used to analyze the parameter variability in the PBTK model during the assessment of predicting ability in population toxicokinetics. The methods for uncertainty analysis are also applicable to variation analysis, and the MCMC is the classic method for variability analysis [95,96,97,98]. In their study on the maximum detection time (MDT) of 1,1-difluoroethane in the blood after inhalation abuse among the adult male population in the United States, Huet et al. [99] introduced variability in huffing pattern and body mass index using the Monte Carlo simulation within the PBTK model, and the results indicated that the MDT of 1,1-difluoroethane in blood after abuse ranged from 7.8 to 15.8 h.

#### 2.4.4. Sensitivity Analysis

The sensitivity analysis reflects the impact of parameter changes on model prediction results and is expressed by sensitivity ratio as Equation (16) [76]:

(20) $$S=\frac{\partial O}{\partial I}\times \frac{I}{O}$$

In Equation (20), S is the sensitivity value representing the percentage change of the corresponding output value when a certain input parameter changes by 1% and other input parameters remain unchanged. O is the output prediction result of the model, I is the input parameter of the model. $\left|S\right|\geq 0.5$ means a highly sensitive parameter; $0.2\leq \left|S\right|<0.5$ means a medium sensitivity parameter; $0.1\leq \left|S\right|<0.2$ means a low sensitivity parameter; $\left|S\right|<0.1$ means an insensitive parameter, and the parameter sensitivity is not significant.

To analyze the interaction effects of parameters on the prediction results, the above sensitivity analysis method is not applicable, and the global sensitivity analysis is suggested. Campolongo and Saltelli proposed the two-step method for global sensitivity analysis. Firstly, the Morris method was utilized for screening the parameters potentially involved in interactions; then, the effects of these interactions were quantified using the extended Fourier amplitude sensitivity test [100], a reliable and efficient method suitable for small sample sizes [101,102]. A high sensitivity value of a certain parameter means that a slight change of the parameter will dramatically affect the prediction results. The parameters with high sensitivity are considered the key factors that should be accurately controlled to improve the prediction accuracy of the models. Jeong et al. [103] used a PBTK approach, coupled with global sensitivity analysis, to predict and evaluate the pharmacokinetic profiles of nafamostat in a virtual healthy population under various dosing regimens, and the results suggested that the hepatic distribution and metabolism had a significant impact on systemic exposure and clearance of nafamostat.

#### 2.4.5. Model Optimization

When the predicted value differs greatly from the measured value, the model structure or parameters may be inappropriate, and model optimization should be performed. In vivo experiments are suggested to clarify the ADME process for a reasonable model structure. For the prediction deviation caused by inappropriate parameters, the uncertainty, variability, and sensitivity analysis can be performed to determine the parameters that greatly impact the prediction value. Then, the parameters are optimized by in vivo, in vitro, and in silico methods to improve the prediction accuracy of the models.

## 3. Applications of Physiologically Based Toxicokinetics (PBTK) in Health Risk Assessment

### 3.1. Exposure Assessment

Forward dosimetry is used to predict the internal dose (tissular chemical concentration) by external dose (exposure chemical concentration) and is called reverse dosimetry and vice versa [104]. The PBTK model establishes a reliable correlation between the external dose and internal dose so that reverse dosimetry is particularly suitable for exposure assessment [105]. It is suitable for practical applications in exposure assessment, such as population-based studies on herbicide fluorine [106]. If the external dose is linear with the internal dose, it can be calculated by the following equation:

(21) $$\begin{array}{c} \mathrm{Exposure}\;\mathrm{dose}=\frac{C}{\left[X\right]}\; \end{array}$$

where C is the measured concentration of the chemical or metabolite in tissues, and [X] is the theoretical value in a biological sample at a simulated exposure dose of one unit. [X] represents the maximum concentration, area under the curve, or other toxicokinetic parameters.

### 3.2. Extrapolation

Compared to classical compartment models and population pharmacokinetic models, the PBTK model, which is based on physiological and anatomical features, is more suitable for extrapolation. Many parameters in the PBTK model cannot be directly obtained through in vivo experiments; therefore, in vitro and in silico assays are necessary. In the PBTK model, various extrapolations such as in vitro-in vivo extrapolation (IVIVE), dose extrapolation, exposure route extrapolation, and interspecific extrapolation are performed to predict toxicokinetic processes across different species and exposure conditions. This approach significantly reduces the reliance on animal experiments while conserving human and material resources, aligning with the “3R” principle of animal ethics.

#### 3.2.1. IVIVE

The in vitro cellular assays used for predicting the in vivo ADME processes of chemicals in IVIVE do not account for active transport, biotransformation, and other factors, leading to uncertainty in this extrapolation method. Poulin et al. [107] highlighted the need for adjustments when applying intracellular free concentrations obtained from in vitro assays to in vivo studies. While novel cell culture methods such as 3D cell culture and coculture provide better simulation of the in vivo state compared to traditional monolayer cell culture, their complexity and cost restrict their utilization for high-throughput screening. In this context, the PBTK model used in IVIVE offers a more accurate and convenient prediction of chemical ADME processes in vivo [108].

The reverse dosimetry approach based on PBTK modeling enables establishing a relationship between in vitro concentrations and human exposure doses, facilitating a dose-response assessment using IVIVE. In their study, Fabian et al. [109] employed the PBTK model to extrapolate the lowest observed effect concentrations of ten environmental endocrine disruptors determined in vitro to the equivalent oral doses in rats and compared them with the lowest observed effect levels (LOEL) values derived from animal studies. Results indicated that predicted LOEL values for six out of ten chemicals were consistent with those obtained from animal experiments within an acceptable range, while the remaining four differed by more than 10-fold. This investigation highlights the potential utility of reverse dosimetry based on the PBTK model for conducting IVIVE analysis, although further improvements in accuracy are needed.

Dejongh et al. [110] developed a PBTK model using data from in vitro assays, such as tissue-blood partition coefficients and liver metabolism parameters. The in vivo neurotoxicity of eight neurotoxicants (benzene, toluene, lindane, acrylamide, parathion/paraoxon, caffeine, diazepam, and phenytoin) was predicted and compared to literature-based in vivo values. The predicted values showed a correlation with the experimental values, with greater accuracy observed for low neurotoxic chemicals (approximately 2-fold deviation) compared to high neurotoxic chemicals (approximately 10-fold deviation). This study suggested the potential application of PBTK models in IVIVE studies. Brinkmann et al. [111] focused on two classes of chemicals, using 7-ethoxyresorufin-O-deethylase and vitellogenin as exposure biomarkers in Oncorhynchus mykiss. The half-effect concentrations (EC50) of these chemicals were determined through both in vitro and in vivo assays. The in vitro EC50 was used as a dose parameter in the PBTK model to predict chemical concentrations in the blood and liver of Oncorhynchus mykiss. Linear correlations were observed between in vitro EC50 and the concentrations of chemicals in the blood and liver, indicating that combining the PBTK model with in vitro assays can accurately predict results from in vivo experiments.

#### 3.2.2. Dose Extrapolation

In toxicology experiments, a high dose exposure is commonly employed; however, actual exposure to xenobiotics typically occurs at low doses. Consequently, dose extrapolation becomes crucial in risk assessment, particularly when transitioning from high to low doses. Simple linear extrapolation often leads to significant deviations due to potential saturation of absorption, metabolism, and excretion during high dose exposure. To address this issue more accurately, the PBTK model incorporates nonlinear equations such as the Michaelis-Menten equation to describe the nonlinear processes of chemicals in vivo. For example, Wang et al. [112] employed the PBTK model to simulate the toxicokinetic of vinyl chloride in the human body after exposure to 100 ppm for 6 h. Subsequently, this model was utilized to predict the exhaled breath concentration of vinyl chloride following exposures of 59 ppm and 261 ppm for 7.5 h. The PBTK model’s predicted values aligned closely with the measured values, thereby demonstrating its precise predictive capability for in vivo concentrations of inhaled vinyl chloride.

#### 3.2.3. Exposure Route Extrapolation

Exposure route extrapolation involves developing a model based on intravenous administration and incorporating absorption processes to extrapolate to non-intravenous routes such as oral, subcutaneous, or inhalation exposure. Traditional extrapolation methods often assume complete absorption in different routes, disregarding factors like the first-pass effect in oral administration. The PBTK model can simulate the absorption process in various exposure routes, offering a more scientifically sound and accurate extrapolation approach.

Gajewska et al. [113] employed a PBTK model to extrapolate the oral no observed adverse effect level (NOAEL) of three cosmetic ingredients (coumarin, hydroquinone, and caffeine) in animal studies to estimate the corresponding oral NOAEL in humans. Based on this, they predicted the concentration in the human body following percutaneous absorption. The findings revealed that for low exposure doses, the oral route did not consistently result in higher in vivo concentrations compared to the percutaneous route.

#### 3.2.4. Interspecific Extrapolation

In toxicological experiments, ethical considerations often require testing on experimental animals, and the findings need to be extrapolated to humans for risk assessment purposes. Traditional extrapolation methods rely on scaling factors such as body weight or body surface area, which fail to account for metabolic differences between species and consequently introduce significant uncertainty. Interspecific extrapolation using the PBTK model allows the accurate prediction of toxicokinetic processes across different species by adjusting species-specific parameters such as physiological and metabolic rate parameters.

The human PBTK model for acrylamide and its metabolite epoxyproacrylamide was developed by Sweeney et al. [114], based on the rat PBTK model, with adjustments made to certain parameters using human data. The model successfully predicted the toxicokinetic processes of these compounds in humans, yielding results consistent with literature measurements. Furthermore, this model was employed to investigate the safety dose of acrylamide in humans.

### 3.3. PBTK of the Special Population

The primary distinction between the special population and the general population lies in the alterations observed in physiological structure and parameters. Consequently, a PBTK model designed for a specific population can be developed. This involves modifying the general population model framework, replacing standard physiological parameters with those specific to the particular population, and integrating distinctive mechanisms related to specific chemicals. Commonly employed PBTK models for special populations encompass three categories: pregnant women and fetuses, children, as well as patients afflicted with hepatic or renal impairment.

For pregnant women and fetuses, they are often modeled together rather than separately in PBTK models. Abduljalil et al. [115] investigated the anatomical, physiological, and metabolic changes occurring in pregnant women throughout gestational weeks. Studies have demonstrated significant alterations in crucial parameters such as cardiac output, protein binding, and metabolic enzyme activity during pregnancy. Moreover, many physiological parameters change non-linearly over time and require description through distinct algorithms. Xia et al. [116] presented a relatively simple structure of the PBTK model for pregnancy, incorporating essential physiological parameters like renal function and CYP450 enzyme system activity. The model was validated using four reference drugs. Wu et al. [117] expanded the PBTK model constructed by Xia et al. by adding physiological parameters such as fetal placenta, fat, and plasma volume for the application in pregnant women. Dallmann et al. [118] provided a comprehensive review of existing PBTK models during pregnancy, characterizing the model structures for pregnant women and fetuses. In this model, the fetus was connected to the mother through the umbilical artery and vein of the placenta. Corley et al. [119] assessed the applicability of PBTK models for pregnant and lactating women in child risk assessment. The PBTK model developed by Lu et al. [120] integrated variations in enzyme activity and accurately predicted the therapeutic doses of three drugs, namely caffeine, metoprolol, and midazolam, which were metabolized by CYP1A2, CYP2D6, and CYP3A4 respectively, in pregnant women. The findings revealed a two-fold increase in caffeine exposure among pregnant women compared to their non-pregnant counterparts.

For children, physiological parameters vary significantly across the different stages of development from infancy to 18-year-old adolescence. Therefore, it is more appropriate to express these parameters as age-related. Edgington et al. [121] summarized changes in relevant physiological parameters in children, such as body weight, height, organ weight, and organ-specific blood flow. They modified adult PBTK models by incorporating age-related adjustments and proposed age-corrected models to predict chemical concentrations in plasma for children at corresponding ages. Kovar et al. [122] developed an adult PBTK model for buprenorphine and norbuprenorphine, and this model was also suitable for children and preterm neonates since it considered age-related changes. Khalil et al. [123] provided a summary of the methodology, application, and limitations of PBTK models for pediatric medication. Generally, the PBTK model proves valuable in simulating and predicting pharmacokinetics after medication administration in children by providing guidance on optimal timing, dosage, and frequency while avoiding ethical concerns. However, there are limitations due to the incompleteness of physiological process descriptions and variability and uncertainty in the population, limiting the clinical application of these models.

In the case of hepatic and renal impairment, the primary impact is on the clearance of chemicals from the body, leading to a slower decrease in plasma concentration. To predict the temporal profile of chemical concentrations in patients with hepatic and renal impairment, it is necessary to adjust physiological parameters affected by liver and kidney damage without modifying the model structure [124]. Compared to a healthy state, parameters such as hepatic and renal blood flow, liver and kidney function, plasma protein binding rate, enzyme activity, and elimination processes are primarily affected. Furthermore, enzyme activity, CL, and biliary excretion are affected during oral exposure in cases of hepatic impairment [125,126]. Additionally, chemicals primarily metabolized by the liver are significantly affected by renal impairment. This condition decreases glomerular filtration rate (GFR), renal tubular secretion, and protein binding and has repercussions on intestinal and hepatic [127]. Strougo et al. [124] proposed a semi-PBTK model for patients with both hepatic and renal insufficiencies to accommodate situations where data availability is limited since full PBTK models necessitate an extensive. Li et al. [128] used the PBTK model to predict the intravenous and oral pharmacokinetics of bisoprolol across multiple dose levels in normal adult human populations and patients with renal impairment. During the model construction process, parameters such as GFR, creatinine clearance rate, age, and tissue blood flow were adjusted to represent patients with impaired renal function.

### 3.4. Metabolic Characteristics and Mechanisms

Complex models can be built based on the ADME processes of chemicals to simulate the effects of transporters, metabolic enzymes, and receptors, thereby predicting the internal dose of specific organs or tissues. Through the integration of TK and toxicodynamic (TD) models, a concentration–effect relationship can be established to aid in investigating the mechanism underlying xenobiotic toxicity. For example, glycyrrhetinic acid (GA) inhibits renal 11β-hydroxysteroid dehydrogenase 2 (11β-HSD 2) and enhances mineralocorticoid receptor activity, leading to pseudo-hyperaldosteronism. Xu et al. [129] developed a human PBTK model of GA and a renal TK/TD model that can describe the effects of 11β-HSD 2 and mineralocorticoid receptors. The biomarkers such as urine cortisol and cortisone concentrations, as well as endpoints including angiotensin II, aldosterone, potassium ions, and sodium ions of GA were predicted using this model. This integrated model can be utilized to determine the safe dosage of GA.

### 3.5. Mixture Risk Assessment

In daily life, exposure to multiple chemicals simultaneously is a common occurrence. Synergistic and antagonistic interactions between these chemicals can either enhance or weaken their toxic effects. Therefore, accurately assessing the risk of chemical mixtures requires considering these interactions rather than simply calculating the total toxic effects through addition or subtraction. The PBTK model can account for the pharmacokinetic characteristics of chemicals and accurately evaluate the toxic effects of mixtures by incorporating mixture interactions. In studies involving multiple mixtures, it is common practice to establish a binary mixture PBTK model and subsequently build upon it to develop a multivariate mixture PBTK model. For instance, Haddad et al. [130] constructed a PBTK model to simulate the toxicokinetics of mixtures composed of benzene (B), toluene (T), ethylbenzene (E), and m-xylene (X) in rats. Initially, pairwise chemical combinations were simulated with optimized parameters obtained from experimental data for B-T, B-E, B-X, T-E, T-X, and E-X. The mechanism of interactions between binary mixtures was determined by evaluating the metabolic inhibition constant Ki for competitive or noncompetitive inhibition scenarios. Subsequently, using this information as a foundation, they developed a comprehensive PBTK model for all four chemicals in the mixture, which effectively predicted their respective toxicokinetic processes.

## 4. Prospects

The PBTK model has undergone gradual development since its initial proposal by Teorell in the 1930s [131], particularly from the 1960s onwards. In the 1980s, it found application in toxicology and became known as the PBTK model. With advancements in computer science and technology, the PBPK/PBTK model has experienced rapid growth and widespread utilization. Its applications encompass chemical risk assessment, new drug development, and generic drug research. Compared to traditional compartmental models, the PBTK model offers enhanced accuracy in dose determination, exposure route analysis, and interspecific extrapolation, rendering it more suitable for health risk assessment purposes. A robust PBTK model should possess four essential characteristics [11]: (1) a physiologically-based structure with appropriate assumptions, (2) accurate mathematical equations representing the model, (3) precise parameter values within the model, and (4) comprehensive evaluation and verification of the model’s performance. However, meeting these characteristics consistently throughout the construction processes of a PBTK model poses inherent challenges:

(1) The PBTK model is based on the understanding of the in vivo ADME processes of chemicals. However, it is challenging to construct a corresponding PBTK model for certain drugs, such as Class 3 and 4 drugs in the Biopharmaceutics Classification System (BCS), traditional Chinese medicine ingredients, heavy metals, etc., due to limited knowledge about their ADME processes.
(2) The parameters required by the PBTK model are typically obtained through in vivo, in vitro, and in silico assays. However, uncertainties may exist regarding parameters measured using in vitro and in silico assays that require validation with further in vivo experimental data. Moreover, special population groups, such as different races, children, pregnant women, obese individuals, and those who are ill, exhibit distinct physiological and ADME characteristics. Therefore, constructing PBTK models for these special groups necessitates extensive experimental research support.
(3) Validating exposure characteristics among different subjects under various exposure conditions is crucial for establishing a reliable PBTK model. Unfortunately, in toxicological studies, the lack of relevant experimental data poses challenges for verifying the accuracy of PBTK models.
(4) Constructing PBTK models can be relatively complex as it requires researchers to possess fundamental knowledge of toxicokinetics, toxicology, physiology, mathematics, and modeling. This complexity limits accessibility to these models.
(5) For mixtures composed of different types of chemicals, it becomes difficult to construct mixed PBTK models due to variations observed during their respective ADME processes within an organism and also because interaction processes between them can become intricate.

To support scientific researchers and risk assessment practitioners, the US Environmental Protection Agency (EPA) [132] and the World Health Organization (WHO) [80] have issued guidance documents on the PBPK/PBTK model in 2006 and 2010, respectively. These documents provide modeling methods, applications in risk assessment, and standardized model description paradigms to facilitate understanding and qualification by regulatory authorities. Since 2014, the FDA organized multiple PBTK workshops, and modeling reports have been submitted to health authorities to optimize clinical trials or seek clinical study waivers [133]. Recognizing the potential of the PBPK/PBTK model in pharmaceutical development, the FDA and the European Medicines Agency (EMA) released guidance documents in 2016. FDA focused on the standard format and structure of the report [105], and EMA issued the submission of supporting documentation to demonstrate model reliability [106]. The Organization for Economic Cooperation and Development (OECD) has issued a guidance document on PBTK models for regulatory purposes concerning chemicals [134]. The OECD not only provides the process and self-check form for constructing PBTK models but also offers 7541 existing PBTK models for 1150 chemicals and 13 typical cases as references.

Future research in PBTK modeling aims to develop more efficient algorithms that reduce simulation time and user-friendly software with simpler interfaces and lower costs. Integration with quantitative pharmacology, systems biology, cellular pharmacokinetics, and other disciplines will expand the capabilities of the PBTK model. Recognition and acceptance by government and regulatory authorities will play a vital role in promoting its application and development. The advancement and utilization of the PBTK model require interdisciplinary collaboration among physiology, biochemistry, pharmacology, toxicology, mathematics, computer science, and software engineering, as well as involvement from government agencies, academia, research institutions, and enterprises. This collective effort will drive the development and application of the PBTK model, including a more complex mixture of PBTK models, PBTK/TD models, and the transition from animal-based testing methods to non-animal alternatives in health risk assessment. However, when utilizing PBTK models, it is crucial to consider various biological variabilities, differences in exposure scenarios, and the accuracy of model parameters to ensure the reliability and precision of the predictions. It is noteworthy that PBTK models predominantly focus on vertebrates, raising concerns about their applicability to other acquisition of corresponding model parameters when constructing PBTK models for non-vertebrate species within the ecosystem present critical considerations. These issues necessitate attention in PBTK models targeting ecological systems and require resolution in future research.

## 5. Conclusions

This review systematically delineates the characteristics of PBTK models, providing a comprehensive overview covering aspects of modeling, application, and prospects. The application of PBTK models has not only deepened our understanding of biological processes within the body but also provided substantial support for fields such as drug exploitation and environmental toxicology. The continuous development and refinement of PBTK models will offer more accurate and reliable tools for health risk assessment.

## Figures and Tables

**Table 1:** The mathematical equations in the PBTK model [15].

Toxicokinetic Process	Equation	
Absorption		
Respiratory tract	$C_{a}=\frac{Q_{p}\cdot C_{inh}+Q_{c}\cdot C_{v}}{Q_{c}+Q_{p}/P_{b}}$	(2)
Percutaneous	$\frac{{dA}_{sk}}{dt}=K_{p}\cdot S\left(C_{air}-\frac{C_{sk}}{P_{s:a}}\right)+Q_{sk}\cdot \left(C_{a}-\frac{C_{sk}}{P_{s:b}}\right)$	(3)
Oral	$\frac{dA_{o}}{dt}=K_{o}\cdot \left(A-A_{o}\right)$	(4)
Intravenous	$C_{v}=\frac{K_{z}+\left(\sum_{t}^{n}Q_{t}\cdot C_{vt}\right)}{Q_{c}}$	(5)
Distribution		
Protein binding	$C_{b}=\frac{n\cdot \beta \cdot K_{d}\cdot C_{f}}{1+K_{d}\cdot C_{f}}$	(6)
Irrigation rate-limiting structure	$\frac{{dA}_{t}}{dt}=Q_{t}\cdot \left(C_{a}-C_{vt}\right)$	(7)
Membrane rate-limiting tissue	$\frac{{dA}_{t}}{dt}={PA}_{t}\cdot \left(C_{vt}-\frac{C_{t}}{P_{t}}\right)$	(8)
Metabolism		
First-order kinetic	$\frac{{dA}_{met}}{dt}=K_{f}\cdot C_{vt}\cdot V_{t}=CL\cdot C_{vt}$	(9)
Second-order kinetic	$\frac{{dA}_{met}}{dt}=K_{s}\cdot C_{vt}\cdot V_{t}\cdot C_{cf}$	(10)
Saturation process	$\frac{{dA}_{met}}{dt}=\frac{V_{max}\cdot C_{vt}}{K_{m}+C_{vt}}$	(11)
Excretion		
Kidney	$\frac{{dA}_{rc}}{dt}=\left(GFR\frac{T_{m}}{K_{t}+C_{p}}\right)\cdot C_{p}$	(12)
Lung	$C_{x}=\left(0.7\cdot \frac{C_{a}}{P_{b}}\right)+\left(0.3\cdot C_{inh}\right)$	(13)

Equation (2): Q_c_, cardiac output; Q_p_, alveolar ventilation; P_b_, blood-air distribution coefficient; C_a_, xenobiotics concentration in arterial blood; C_inh_, xenobiotics concentration in inhaled gas; C_v_, xenobiotics concentration in mixed venous blood. Equation (3): A_sk_, total amount of skin xenobiotics exposed; t, time; K_p_, skin permeability coefficient; S, skin exposure area; C_air_, concentration of xenobiotics in the air; C_sk_, concentration of exposed skin xenobiotics; P_s:a,_ skin-air distribution coefficient; Q_sk_, skin blood flow; P_s:b_, skin-blood partition coefficient. Equation (4): A_o_, total amount of xenobiotics absorbed; K_o_, oral absorption rate constant; A, oral exposure dose. Equation (5): K_z_, intravenous administration rate; Q_t_, blood flow in “t” chamber; C_vt_, xenobiotics concentration in venous blood of outflow chamber “t”. Equation (6): C_b_, binding xenobiotics concentration; n∙β, maximum binding rate; K_d_, dissociation constant; C_f_, free xenobiotics concentration. Equation (7): A_t_, total amount of xenobiotics in “t” chamber. Equation (8): PA_t_, mass transfer coefficient; P_t_, organ/tissue–blood allocation coefficient. Equation (9): A_met_, total amount of xenobiotics metabolism; K_f_, first-order metabolic rate constant; V_t_, “t” chamber volume; CL, clearance. Equation (10): K_s_, second-order me tabolic rate constant; C_cf_, cofactor concentration. Equation (11): V_max_, maximum velocity of enzyme-catalysis; K_m_, Michaelis constant. Equation (12): A_rc_, total amount of xenobiotics in kidney; GFR, glomerular filtration rate; T_m_, apparent maximum transport rate of the carrier system; K_t_, apparent Mieman constant; C_p_, xenobiotics concentration in plasma. Equation (13): C_x_, xenobiotics concentration in exhaled gas.

**Table 2:** The physiological, physicochemical, and biochemical parameters in the PBTK model.

	Parameter	Data Source
Physiological parameter	Body weight Cardiac output Blood flow to organ or tissue Volume of an organ or tissue Alveolar ventilation	Literature In vivo experiment.
Physicochemical parameter	Blood-air distribution coefficient Tissue-blood distribution coefficient	Literature In vivo experiment In vitro experiment In silico prediction
Biochemical parameter	Maximum velocity Michaelis constant First-order rate constant Second-order rate constant	Literature In vivo experiment In vitro experiment In silico prediction

For the parameters unable to be obtained, the accessible data can be fixed in the PBTK model, and added data can be measured, thus simulating and obtaining uncertain data.

## Data Availability

All data are included in this manuscript.
